# Sickle cell disease: a neglected chronic disease of increasing global health importance

**DOI:** 10.1136/archdischild-2013-303773

**Published:** 2014-09-19

**Authors:** Subarna Chakravorty, Thomas N Williams

**Affiliations:** 1 Department of Medicine, Imperial College, London, UK; 2 KEMRI-Wellcome Trust Research Programme, Kilifi, Kenya

**Keywords:** Haematology, sickle cell disease

## Abstract

Sickle cell disease (SCD) is a single gene disorder causing a debilitating systemic syndrome characterised by chronic anaemia, acute painful episodes, organ infarction and chronic organ damage and by a significant reduction in life expectancy. The origin of SCD lies in the malarial regions of the tropics where carriers are protected against death from malaria and hence enjoy an evolutionary advantage. More recently, population migration has meant that SCD now has a worldwide distribution and that a substantial number of children are born with the condition in higher-income areas, including large parts of Europe and North and South America. Newborn screening, systematic clinical follow-up and prevention of sepsis and organ damage have led to an increased life expectancy among people with SCD in many such countries; however, in resource-limited settings where the majority continue to be born, most affected children continue to die in early childhood, usually undiagnosed, due to the lack of effective programmes for its early detection and treatment. As new therapies emerge, potentially leading to disease amelioration or cure, it is of paramount importance that the significant burden of SCD in resource-poor countries is properly recognised.

## Introduction

Sickle haemoglobin (HbS) is a structural variant of normal adult haemoglobin (HbA) caused by a mutation in the *HBB* gene that leads to the substitution of valine for glutamic acid at position 6 of the β-globin subunit (β^S^) of the haemoglobin molecule.^1^ The term ‘sickle cell disease’ (SCD) refers to any condition in which the production of HbS leads to pathophysiological consequences. The most common form (>70% of SCD worldwide)^2^ results from the homozygous inheritance of the β^S^-mutation and is usually referred to as either ‘SCD SS’ or as ‘sickle cell anaemia’ (SCA). However, SCD can also result from the inheritance of β^S^ in combination with a wide range of other *HBB* mutations, the two most common being a second structural β-globin variant β^C^ (SCD SC)^3^ and one of the many β-thalassaemia mutations that lead to the reduced production of normal β-globin (SCD S/β-thalassaemia).^4^ SCD SS is the most severe form of SCD and, consequently, is the main focus of the current review.

## Historical perspective

SCD was first described in the Western medical literature by the American physician James Herrick who reported the presence of ‘peculiar elongated and sickle-shaped red blood corpuscles’ in the blood film of a Grenadan student with a history of leg ulcers, shortness of breath and jaundice.^5^ Pauling and Itano^6^ established the fact that SCD was a molecular disease almost 50 years later while in the decades that followed, scientific advances led to descriptions of the structure of the HbS molecule,^7^ molecular basis of the sickling phenomenon,^1^ cloning and sequencing of the β-globin gene, development of molecular diagnostic methods^8^ and establishment of prenatal diagnosis.^9^

In parallel with such advances, significant progress was made towards improving clinical outcomes among those born with SCD during the 1970s and 1980s, before which very few affected subjects survived beyond 10 years.^10^ In response to reports of poor funding for SCD research, a series of comprehensive SCD centres were created in the USA during the 1970s,^11^ and by 1994 the median age of death had risen to 48 and 42 years in women and men, respectively.^12^ ^13^ Following the introduction of newborn screening programmes in cohorts in the USA,^14^ Jamaica^15^ and the UK,^16^ and the gradual introduction of a broad range of life-saving measures (including penicillin prophylaxis, vaccination for common bacterial diseases, training of parents to detect splenic sequestration events and provision of disease-modifying treatment with hydroxycarbamide), in the US cohort overall survival to 18 years had risen to 85% by 2004^14^ and to 96% by 2010,^17^ while in the London cohort overall survival to 16 years was almost universal by 2007.^16^ Nevertheless, despite these dramatic improvements, the outcome for adolescents and adults with SCD remains disappointing. In a recent US study, mortality among patients aged 20–25 years was twice that of patients aged 15–19 years, highlighting the importance of the transition from paediatric to adult services.^18^

## The burden and global distribution of SCD

The β^S^-mutation is the archetypal example of natural selection in humans. Heterozygotes, whose red blood cells contain both HbA and HbS, are so strongly protected from malaria^19^ ^20^ that the global distribution and the frequency of the β^S^-mutation a mutation now strongly reflects the historic incidence of death from malaria.^21^ Nevertheless, despite the extraordinary protection that β^S^-carriers enjoy from malaria, there are few places where the carrier frequencies exceed 25% because the rise of the mutation in populations above that level has been kept in check by the profound disadvantage conferred by homozygosity.^22^ Despite the fact that SCD originates in the malaria-endemic world, population migration during the last few hundreds of years, first through the slave trade and more recently for economic and work-related reasons, means that substantial numbers of children with SCD are now being born in high-income countries, particularly in the larger cities in Europe and North America.^23^ No global data regarding the precise numbers of children born with SCD exist because, in contrast to Europe and North America, newborn screening for SCD is not available in most resource-poor countries with the highest predicted burdens; however, on the basis of data on carrier frequencies and global birth rates, we recently estimated that around 312 000 children are born each year with SCD SS, a figure that includes approximately 300 births in the UK and almost 3000 in the USA.^24^ Given the more limited availability of detailed contemporary allele frequency data for haemoglobin C (HbC) and the β-thalassaemias, it is more difficult to estimate the numbers born with other forms of SCD, but they probably total a further 50 000–100 000 births per year.^2^

Despite the numbers born with SCD in resource-poor countries, remarkably, few detailed studies have described the clinical course and complications of the disease in that context. Even today, the majority of those born with the disease in Africa, where more than 80% of affected births occur, die undiagnosed in early childhood,^22^ presumably from preventable causes that include invasive bacterial diseases,^25^ malaria^26^ and severe acute anaemia. As a consequence, in many parts of sub-Saharan Africa, SCD is probably responsible for up to 6% of all child deaths,^2^ ^27^ a situation that must be addressed if recent improvements in overall child survival are to be consolidated. Because so little is known about the clinical course and natural history of SCD in resource-poor countries, the majority of this review is focused on data from Europe and North America, where most detailed studies have been conducted.

## Diagnosis and pathophysiology

The diagnosis of SCD relies on the analysis of haemoglobin, most commonly using either protein electrophoresis or high-performance liquid chromatography. Subjects with the most common form of SCD, SCD SS, produce no HbA, but predominantly produce HbS along with variable amounts of haemoglobin F (HbF) and haemoglobin A_2_ (HbA_2_), while those with SCD SC produce mainly HbS and HbC. DNA-based methods are commonly used to confirm the diagnosis of SCD in more complicated cases.^28^

Since SCD was first described a century ago, a great deal has been learnt about its pathophysiological consequences. Under conditions of hypoxia, acidity and cellular dehydration, the polymerisation of HbS within erythrocytes leads to their deformation into the characteristic ‘sickle’ shape. In dynamic interaction with the vascular endothelium, this sickling leads to episodic microvascular occlusion, ischaemia and reperfusion,^29^ vascular and inflammatory stress, and increased expression of vascular oxidases, inflammatory cytokines and adhesion molecules.^29^ In addition, chronic haemolysis results in anaemia, hypoxia, cholelithiasis, fatigue, exercise intolerability, hypercoagulability^30^ and vasculopathy,^31^ which lead in turn to endothelial nitric oxide depletion, development of pulmonary hypertension^32^ and ischaemic strokes.^33^ Recent work in transgenic sickle mice has highlighted the central role played by hypoxia in generating multi-organ damage by increased adenosine signalling via the G-protein coupled adenosine receptor ADORA2B.^34^

The pathophysiology of pain in SCD remains poorly understood.^35^ Nociceptive stimuli generated from cellular responses to vaso-occlusion, tissue infarction, inflammation and ischaemia-reperfusion injury activate receptors in the peripheral sensory nerves. However, neuropathic pain and increased sensitisation to mechanical touch have also been frequently noted, the latter being recently characterised as driven by increased primary afferent input to the central nervous system by the transient receptor potential vanilloid-1 channel in transgenic sickle mice.^36^

## Clinical features

The clinical features of SCD, described through multiple studies conducted in high-income populations in Europe and North America, are defined by chronic anaemia, sepsis, haemolysis and recurrent acute vaso-occlusive crises. The last are characterised by pain and a systemic inflammatory response that may be severe, episodic and unpredictable. Some of the more common acute clinical and laboratory features of SCD are summarised in table 1, along with descriptions of current approaches to their management. Although this list would almost certainly look very different among children with SCD in low-income countries who are often exposed to malaria, geo-helminth infections, undernutrition and variable standards of care, this is beyond the scope of the current review.

**Table 1:** Common clinical presentations of SCD

Clinical presentation	Symptoms	Laboratory findings	Treatment
Painful crisis	Pain, localised swelling, fever	Low Hb, high reticulocyte count, high LDH, high bilirubin, high CRP	Hydration Analgesia Antibiotics
Acute chest syndrome	Chest pain, fever, hypoxia, cough; may progress from painful crisis elsewhere	Low Hb, high reticulocyte count, high LDH, pulmonary infiltrates on CXR	Respiratory support, antibiotics, red cell exchange transfusion
Bacterial sepsis	Pain, localised swelling, fever	Low Hb, high reticulocyte count, high LDH, high CRP, positive cultures	Hydration Analgesia Antibiotics
Sequestration crisis	Pain, severe pallor, hepatomegaly or splenomegaly	Low Hb, high reticulocyte count	Urgent red cell transfusion, pain relief
Aplastic crisis	Pallor	Low Hb, low reticulocyte count, parvovirus B19 +ve	Urgent red cell transfusion
Acute ischaemic stroke	Hemiplegia, altered consciousness, seizures	MRI brain with characteristic findings	Urgent red cell exchange transfusion aim to reduce HbS to <30%
Girdle syndrome	Abdominal pain and distension, reduced or absent bowel sounds, pallor, fever	AXR may show dilated bowel loops. Low Hb, high reticulocyte count, high CRP	Nil by mouth, NG tube on free drainage, broad spectrum antibiotics with anaerobic cover, red cell exchange transfusion, surgical review
Priapism	Painful, persistent erection		Hydration, pain relief, urgent urology review and intervention: red cell exchange transfusion

AXR, abdominal x-ray; CRP, C-reactive protein; CXR, chest x-ray; Hb, haemoglobin; HbS, sickle haemoglobin; LDH, lactate dehydrogenase; MRI, magnetic resonance imaging; NG, naso-gastric; SCD, sickle cell disease.

## Vaso-occlusive crises and bone disease

Painful vaso-occlusive crisis due to bony infarction is the commonest cause for hospital admissions. Infants may present with dactylitis or bony infarction of digits, irritability and swelling of fingers or toes. Infarction can affect any bone or joint and may mimic osteomyelitis.^37^ Avascular necrosis—the result of recurrent vaso-occlusion and infarction of the articular surfaces and heads of long bones—is found in 12%–15% of children with SCA^38^ ^39^ and has been associated with both high haematocrit and the concomitant presence of α-thalassaemia.^40^ ^41^ Osteomyelitis and septic arthritis, most commonly due to *Salmonella* spp, *Staphylococcus aureus* and Gram negative enteric bacilli,^42^ are also common, a cumulative incidence of 12% having been reported in one paediatric cohort in metropolitan France.^43^ Osteopaenia and osteoporosis are frequent findings in SCD and patients may suffer from chronic back pain as a result of vertebral collapse.^38^

## Acute chest syndrome

Acute chest syndrome (ACS) is the second most common cause of hospitalisation and is characterised by intrapulmonary ischaemia and infarction, systemic hypoxia and pulmonary infiltrates on chest radiography.^44^ Community-acquired pneumonias and fat embolism from bone marrow necrosis have been implicated in its pathogenesis. In a recent study, 50% of paediatric and adolescent patients with SCD reported acute pulmonary events during a median follow-up of 21 months. Children with asthma, a major cause of morbidity in SCD, suffer twice as many episodes of ACS as those without^45^ while other risk factors include a high white cell count, and a high tricuspid-regurgitant jet velocity (TRV).^46^ While raised TRVs have been associated with mortality in adults,^32^ no such correlation has been reported in children; nevertheless, in one recent study, a gradual decline in exercise tolerance was noted in follow-up of children with elevated TRVs, suggesting that tricuspid valve disease may well progress throughout childhood.^47^

## Bacterial sepsis

Evidence of reduced splenic function is evident from early childhood. Functional asplenia is the norm by 6 months to 3 years of age^48^ ^49^ and leads to an increased susceptibility to infections, particularly those caused by encapsulated bacteria and malaria.^25^ ^26^ ^50^ ^51^ In high-income countries, mortality from sepsis was greatly reduced following the introduction of newborn screening and the early implementation of penicillin prophylaxis, while further improvements in survival and reductions in documented blood stream infections were subsequently achieved following the introduction of *Haemophilus influenza*e and *Streptococcus pneumoniae* vaccines.^17^ Nevertheless, the combination of suboptimal compliance and resistance to penicillin prophylaxis, non-vaccine serotypes of *S. pneumoniae*, and hyposplenism means that even today children with SCD remain at increased risk of bacterial infections.^52^

## Sequestration crises

Splenic sequestration is defined as an acute enlargement of the spleen with a drop in haemoglobin of at least 2 g/dL from baseline and a normal or raised reticulocyte count.^53^ In severe cases, it may result in hypovolemic shock and death in a matter of hours. Splenic sequestration can occur as early as 3 months of age but is rarely seen beyond the age of 6 years. Recurrence can occur in up to 50% of children.^54^ Prompt transfusion can be life saving.^55^ Hepatic sequestration is a rare but severe complication of SCD caused by the obstruction of hepatic sinusoidal blood flow by sickled erythrocytes and is characterised by painful hepatomegaly, anaemia and reticulocytosis.^56^ Treatment is supportive, with fluids and analgesia along with early red cell exchange transfusions.^57^

## Ischaemic strokes and silent infarcts

SCD confers a higher risk of childhood stroke than any other paediatric disease. In all, 11% of patients with SCD develop an overt stroke by the age of 20 years, increasing to 24% by the age of 45 years. The risk of first stroke is highest in the first decade of life, being 1.02% per year between 2 and 5 years.^58^ Two-thirds of children with a history of stroke will develop a second stroke within the first 2–3 years of an initial event.^59^ The risk of stroke can be determined by measuring blood velocities in the middle cerebral and internal carotid arteries by transcranial Doppler (TCD) ultrasonography. In the Stroke Prevention in Sickle Cell Anaemia (STOP) trial, the risk of stroke among children with high TCD velocities was reduced by 90% by maintaining HbS concentrations at <30% through regular blood transfusions.^60^ Children developed further strokes when transfusion therapy was stopped^61^ or treatment was switched to hydroxycarbamide,^62^ highlighting the need for life-long transfusion in this patient group. Silent cerebral infarcts (SCIs) on MRI scanning are common in asymptomatic patients with SCD and are associated with neurocognitive impairment, reduced academic achievement and stroke progression. Regular blood transfusion in children with SCI has recently been shown to reduce the incidence of SCI^63^ and monitoring for worsening intellectual abilities is important.^64^

## Girdle syndrome and priapism

Severe abdominal pain, often unresponsive to analgesia and associated with intestinal ileus and acute ischaemic colitis, is termed ‘girdle syndrome’ owing to the circumferential distribution of pain. A high index of suspicion and early implementation of supportive therapy (including emergency red cell exchange transfusion, analgesia and fluids) may prevent irreversible ischaemic damage to the gut.^65^ Priapism, defined as prolonged penile erection lasting >4 h, is a urological emergency and can result in fibrosis of the corpora cavernosa and permanent erectile dysfunction if not treated early. SCD is the commonest cause of priapism in children and is thought to be caused by chronic nitric oxide depletion within the penile vasculature due to chronic intravascular haemolysis, aberrant G-protein signalling, smooth muscle hypoxia, acidosis and impaired smooth muscle contraction.^66^ Early intervention in the form of red cell exchange transfusion and surgical decompression of the corpora is essential. Recurrent, ‘stuttering’ priapism may be treated by hydroxycarbamide, chronic red cell exchange transfusions or sildenafil.^67^

## Standards of treatment

Universal or targeted newborn screening programmes, implementation of simple treatments such as vaccination and antibiotic prophylaxis, regular follow-up in specialist clinics and improved parental education have together led to major reductions in the early mortality from SCD in high- and middle-income countries. For example, simply teaching parents how to palpate their children's spleens led to a 90% reduction in mortality from splenic sequestration crises in Jamaica.^55^ Nevertheless, despite such encouraging advances, the overall outcome of patients with SCD remains poor. The recent UK National Confidential Enquiry into Patient Outcome and Death for haemoglobinopathies^68^ revealed a significant inequity of specialist care in the country and the lack of adequate knowledge of haemoglobinopathies within the medical community, and recommended the establishment of a national database to capture information regarding prevalence, therapy and adverse events of SCD.

A programme of universal screening for SCD was implemented in England in 2001 and was subsequently rolled out to Scotland and Wales^28^ through which approximately 300 births and 17 000 carriers are detected each year.^69^ The UK National Standards for the Treatment of SCD in Children^70^ highlights the need for coordinating care between the screening service, primary care and local and specialist haemoglobinopathy teams, and mandates the prescription of penicillin to children by 6 months of age along with additional polysaccharide antigen vaccination for *S. pneumoniae*, and the provision of annual TCD monitoring to children over 2 years.

Adequate and prompt management of the acute complications of SCD remains the mainstay of clinical care. While the treatments of common complications are summarised in table 1, pain relief, hydration, aggressive treatment of sepsis and blood transfusions remain central to acute management.

## Specific therapies for SCD

### Hydroxycarbamide

Hydroxycarbamide (or hydroxyurea) remains the only agent that has been proven to reduce the number of episodes of painful crises, ACS and hospitalisations in randomised control trials in adults,^71^ school-age children^72^ and infants^73^ with SCD. Despite its well known beneficial effects and excellent long-term toxicity profile,^74^ utilisation remains suboptimal due to user- and prescriber-related uncertainties regarding toxicities, monitoring and efficacy.^75^

### Blood transfusion

A number of observational and randomised controlled trials have established the pivotal role of transfusion therapy in the management of SCD, most notably in primary stroke prevention^60^ ^61^ and through improved oxygenation in ACS.^76^ Secondary analysis of two large paediatric randomised control trials, namely, the Stroke Prevention (STOP) and Stoke With Transfusions Changing to Hydroxyurea (SWiTCH) trials, indicated that transfusion therapy was more effective in reducing the incidence of painful crises and ACS than either standard supportive care or hydroxycarbamide.^77^ ^78^ Nevertheless, despite its well-recognised benefits, chronic transfusion therapy can result in iron overload (leading to organ damage and requiring additional iron chelation therapy), alloimmunsation, transfusion-acquired infections, venous access-related issues such as thrombosis and line-related sepsis and loss of work and schooling.^79^ Coupled with the fact that the total economic costs of chronic transfusion therapy far exceeds those of hydroxycarbamide treatment,^80^ ^81^ transfusion therapy is mainly reserved for specific indications such as stroke risk reduction, renal failure or recurrent painful crises that are less responsive to treatment with hydroxycarbamide.

### Allogeneic HSCT and gene therapy

Allogeneic haemopoietic stem cell transplant (HSCT) is the only curative treatment for SCD and is successful in 85%–90% of patients.^82^ Transplantation offers disease-free survival and stabilisation of neurological lesions. Nevertheless, the fine balance between the benefits and risks of treatment, including long-term toxicities such as infertility and endocrinopathies,^83^ the variable and unpredictable severity of SCD and the low availability of specialist services mean that HSCT is generally reserved for the most seriously affected patients. Table 2 outlines current indications for HSCT in SCD in the UK. Gene therapy has been in development for a number of years and aims to abrogate SCD-related symptoms by manipulation of haemopoietic stem cells, either by viral vector-mediated insertion of a functional β globin gene or by various gene-editing techniques that reduce intracellular sickling by enhanced production of HbF. Phase I studies of gene therapy have recently begun in several centres in the USA and Europe.^84^ ^85^

**Table 2:** HSCT indications in SCD (ebmt.org/Contents/Resources/Library/EBMTESHhandbook)

One of:	Neurological deficit due to stroke or subarachnoid haemorrhage
	Recurrent acute chest syndrome not responding to 6-month course of hydroxycarbamide
	Recurrent vaso-occlusive crises not responding to 6-month course of hydroxycarbamide
AND	<16 years
AND	HLA-identical matched related donor available
Candidates who may be considered for HSCT in special circumstances:	▸ Problems relating to future medical care e.g. unavailability of adequately screened blood products ▸ SCD relapsing after previous HSCT ▸ Transfusion-dependent S/β^0^ thalassaemia ▸ Adults aged 17–35 years (as part of clinical trial)

HLA, human leukocyte antigen; HSCT, haemopoietic stem cell transplant; SCD, sickle cell disease.

## Future perspectives

In the short term, refining the indications for access to known effective treatments is a major priority; for example, the accumulated data on hydroxycarbamide suggest that the benefits of the drug outweigh the risks in the vast majority of patients and that access to hydroxycarbamide therapy should be available to all who want it. At the same time, new therapies targeting specific mechanisms of HbF induction, endothelial dysfunction, pain management, organ damage and gene therapy are under intense research scrutiny.^86^ Nevertheless, improving the profile of SCD as a major health problem in Africa and India, including the introduction of newborn screening programmes and the improved provision of even the most basic of medical care, will benefit the greatest number of patients with SCD worldwide. A good starting point would be the collection of more detailed and up-to-date data regarding the expected birth frequencies and outcomes of SCD in these regions at scales that will be meaningful to health planners responsible for making such decisions, while estimates of the economic costs and benefits of improved care are also needed. Such data will be most influential if the end-users are involved from the outset. At the same time, improved advocacy for SCD is needed at every level including increased education about SCD in schools and colleges in affected communities, increased involvement of patient-support groups and influential groups such as celebrities, politicians, funders and health agencies internationally. Finally, the development of cheap, reliable point of care methods for the diagnosis of SCD, akin to those developed for other diseases of poverty such as HIV and malaria, could be transformative at many different levels. Translation of research findings to clinical practice in improving patient outcomes worldwide remains the greatest challenge.
